# Plant Communities Rather than Soil Properties Structure Arbuscular Mycorrhizal Fungal Communities along Primary Succession on a Mine Spoil

**DOI:** 10.3389/fmicb.2017.00719

**Published:** 2017-04-20

**Authors:** Claudia Krüger, Petr Kohout, Martina Janoušková, David Püschel, Jan Frouz, Jana Rydlová

**Affiliations:** ^1^Institute of Botany, Academy of Sciences of the Czech RepublicPrůhonice, Czechia; ^2^Faculty of Science, Charles UniversityPrague, Czechia; ^3^Institute of Microbiology, Academy of Sciences of the Czech RepublicPrague, Czechia

**Keywords:** biodiversity, community ecology, fungal and plant succession, ecosystem development, Glomeromycota, mycorrhiza

## Abstract

Arbuscular mycorrhizal fungal (AMF) community assembly during primary succession has so far received little attention. It remains therefore unclear, which of the factors, driving AMF community composition, are important during ecosystem development. We addressed this question on a large spoil heap, which provides a mosaic of sites in different successional stages under different managements. We selected 24 sites of *c.* 12, 20, 30, or 50 years in age, including sites with spontaneously developing vegetation and sites reclaimed by alder plantations. On each site, we sampled twice a year roots of the perennial rhizomatous grass *Calamagrostis epigejos* (Poaceae) to determine AMF root colonization and diversity (using 454-sequencing), determined the soil chemical properties and composition of plant communities. AMF taxa richness was unaffected by site age, but AMF composition variation increased along the chronosequences. AMF communities were unaffected by soil chemistry, but related to the composition of neighboring plant communities of the sampled *C. epigejos* plants. In contrast, the plant communities of the sites were more distinctively structured than the AMF communities along the four successional stages. We conclude that AMF and plant community successions respond to different factors. AMF communities seem to be influenced by biotic rather than by abiotic factors and to diverge with successional age.

## Introduction

Primary succession is generally characterized by soil development and changes in vegetation structure (Odum, 1969). In contrast to successional dynamics of plants, primary succession of mycorrhizal fungi is still poorly understood. Despite early studies describing occurrence of mycorrhizal fungi during succession (Allen et al., 1987, 1992; Warner et al., 1987), the development of their communities along succession has so far received relatively little attention (Dickie et al., 2013). Because of the strong connection between mycorrhizal fungi, plant, and soil, mycorrhizal fungi may play a significant role in ecosystem development.

Arbuscular mycorrhiza is the most common mycorrhizal type known from *c.* 74% of Angiosperm species (Brundrett, 2009) and plays a crucial role in terrestrial ecosystems. All arbuscular mycorrhizal fungi (AMF) belong to the phylum Glomeromycota (Schüßler et al., 2001), which contains *c.* 250 species. Establishment and assembly of AMF communities during primary succession largely depend on AMF dispersal ability to newly exposed land. Studies focused on AMF colonization in pioneer plants show that AMF propagules are abundant at early successional stages of ecosystem development (Püschel et al., 2008; Rydlová et al., 2008). However, it remains unclear whether these pioneer communities are formed by random filtering from local AMF species pools or by immigration of pre-adapted pioneer AMF species with a ruderal life style (Chagnon et al., 2013). If the first was true, high stochasticity in AMF community assembly would be expected, resulting in higher variability (larger composition turnover, Anderson et al., 2011) of the AMF communities in the earliest successional stages (Martínez-García et al., 2015). On the contrary, if early successional sites were predominantly colonized by AMF species with a ruderal life style, composition turnover would not be expected to change during primary succession.

As root-associated mutualistic symbionts, AMF mediate nutrient flow from the soil to the host plant in exchange for assimilated carbon (Smith and Read, 2008). Because of the tight coupling of the AMF life cycle with their host plants, AMF communities are strongly influenced by the identity of host plant species (e.g., Vandenkoornhuyse et al., 2002; Sýkorová et al., 2007). AMF species richness can therefore be expected to increase during primary succession due to increasing plant richness (Walker and Del Moral, 2003), but evidence for that is not consistent (Dickie et al., 2013). As soil dwelling organisms, AMF communities might be affected by changes in soil chemistry during the vegetation development. Particularly pH and macronutrient availability seem to be key deterministic factors governing the richness and composition of AMF communities (Lekberg et al., 2007; Fitzsimons et al., 2008). A number of studies consistently show changes in AMF community composition during ecosystem development (e.g., Oehl et al., 2011; Bennett et al., 2013; Sikes et al., 2014; Krüger et al., 2015; Martínez-García et al., 2015). However, the drivers responsible for the observed differences remain unclear. In their conceptual paper, Zobel and Öpik (2014) proposed that the composition of AMF communities during ecosystem development was driven by host plant–fungus interactions (‘Passenger/Driver hypotheses’ as previously proposed by Hart et al., 2001), rather than abiotic habitat conditions such as soil chemistry or climate (‘Habitat filtering hypothesis’), which become more important in structuring AMF communities at late successional stages.

Indeed, a recently published study showed that host plant identity was a much stronger predictor of root-colonizing AMF communities during ecosystem development than site age, i.e., the response of AMF communities to ecosystem development was mostly associated with plant community changes (Martínez-García et al., 2015). Besides host plant identity, the species richness and community composition of root-colonizing AMF communities is also influenced by neighboring plants (Mummey et al., 2005; Hausmann and Hawkes, 2009; Kohout et al., 2015). The neighborhood of other plant species with distinct AMF communities in roots can both increase the species richness of the root community and induce shifts in AMF community composition of a target plant species (Mummey et al., 2005; Meadow and Zabinski, 2012), and can be as important as the identity of the host plant in structuring AMF communities (Hausmann and Hawkes, 2009). This influence may be exerted by non-random assembling of AMF communities in the roots of different plant species (Davison et al., 2012) as well as by allelopathy via the synthesis of antifungal secondary metabolites (Stinson et al., 2006; Becklin et al., 2012a). Thus, plant succession may influence also AMF communities associated with generalist plants species that persist in the primary successional ecosystems throughout several successional stages.

Primary succession naturally occurs in environments where new substrates are deposited such as glacier forefronts or lava beds. Primary succession also occurs on spoil heaps of spoil material after mining activities (Prach et al., 2013). Spoil material excavated from great depth (hundreds of meters bellow surface) has typically extremely low biological activity (Gould et al., 1996; Frouz et al., 2001; Elhottová et al., 2006). Although ecosystems on man-made sites are usually of shorter history than most natural chronosequences and lack later stages of ecosystem development, understanding the factors that influence primary succession in these areas is important for their re-integration into the landscape. This is also because these man-made sites are often reclaimed with woody plants to accelerate the processes of soil and vegetation development (Frouz et al., 2001, 2007; Abakumov et al., 2013). Factors influencing primary succession at the reclaimed sites, including succession of AMF communities, are therefore even more complex, including the diversity and identity of the planted species and their management.

The main aim of our study was to describe changes in the AMF communities of one generalist host plant during primary succession, and to determine whether they can be related to changes in soil chemistry or to changes in plant community composition, possibly also influenced by reclamation. For this purpose, we chose a unique primary successional chronosequence on a large spoil heap in the western part of Czechia, covered by a mosaic of different successional stages under two different managements (spontaneously developing and reclaimed). In this system, the AM host *Calamagrostis epigejos* can be found throughout the whole chronosequence spanning about 50 years at both unmanaged and reclaimed sites.

We expected that:

(i) The early stage AMF communities became established by random filtering and variation in AMF community composition would therefore decrease during the primary succession.
(ii) The number and structure of neighboring plants rather than soil chemistry would be related to the variability in AMF community composition in *C. epigejos* roots.
(iii) Sites reclaimed by *Alnus glutinosa* plantations would harbor different AMF communities than spontaneously developing sites of the same age.

## Materials and Methods

### Site Description

**FIGURE 1:**
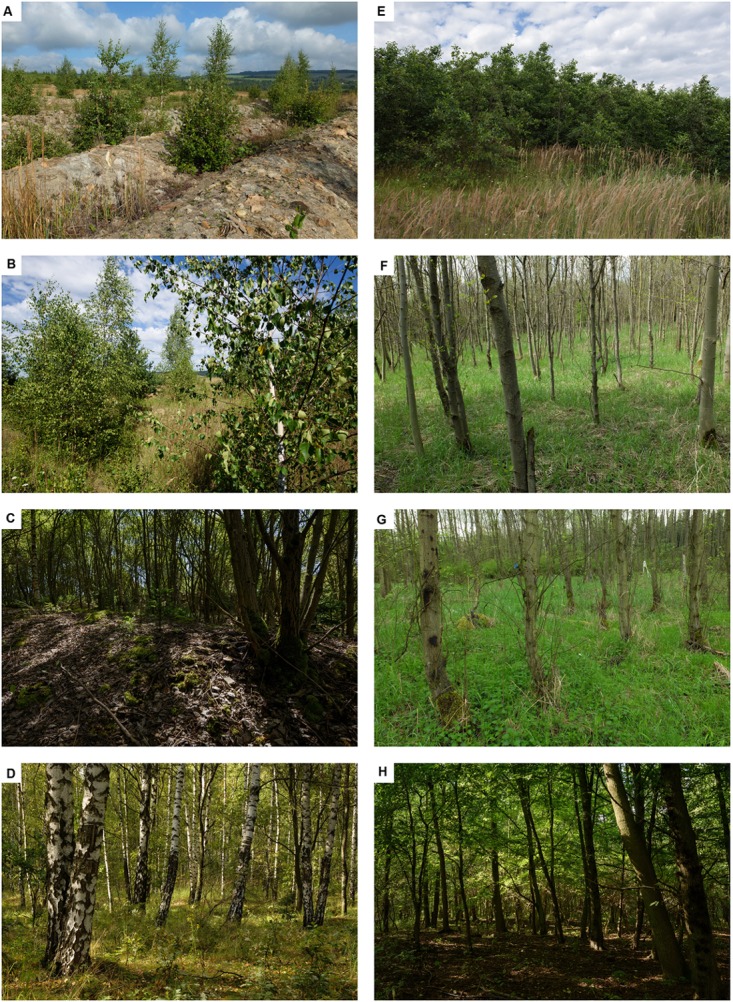
**Illustrative pictures of the sampling sites:** **(A–D)** spontaneously developing chronosequence; **(E–H)** reclaimed chronosequence; **(A,E)** 12-year-old; **(B,F)** 20-year-old stage; **(C,G)** 30-year-old; **(D,H)** 50-year-old.

The studied spoil heap in the Sokolov brown-coal mining district (NW Bohemia, Czechia, 50.2446056N, 12.6741808E) covers an area of *c.* 2.5 km × 10 km and is situated at 500–600 m a.s.l. The mean annual precipitation in the study region is 650 mm and median annual temperature reaches 6.8°C. The spoil heap is composed of deposits of tertiary clays (mainly kaolinite, illite, and montmorillonite) that formed the overburden layers of the exploited coal seam (Křibek et al., 1998) and encompasses patches of reclaimed land as well as land left to spontaneous plant succession. The clay substrates at the sites with spontaneous succession were not rearranged after heaping and become first colonized by herbs and grasses, predominantly *C. epigejos* (L.) Roth (Frouz et al., 2008). The first trees (mainly birches – *Betula pendula* Roth. and willows – *Salix caprea* L.) appear already at the youngest sites and visually dominate the 20-year-old sites. After 30 years, *S. caprea* clearly dominates and creates a closed dense canopy that shades nearly the entire soil surface. Grasses and herbs occur only sporadically at the 30-year-old sites. Subsequently, willows almost completely vanish, and the 50-year-old forest is mainly composed of birches with a dense understory of herbs and grasses (**Figure 1**). The changes in vegetation are accompanied by pedogenesis: At 30-year old sites, the original substrate is covered by a thick fermentation layer of mainly *S. caprea* litter, which becomes gradually transformed into a humus layer, which is characteristic for the 50-year-old sites (Frouz et al., 2008; Rydlová et al., 2016).

The reclaimed sites were leveled by earthmoving machinery and planted with alders [*Alnus glutinosa* (L.) Gaertn.] with density of 10,000 trees per hectare. Alder density gradually decreases with site age, either spontaneously or due to forestry interventions. Tree biomass on reclaimed sites is then lower or equivalent to spontaneously developing sites after ca 25 and 45 years, respectively (Frouz et al., 2015). Woody canopy was not found to significantly differ between the spontaneous and reclaimed sites, probably due to highly variable canopy development within the succession series (Frouz et al., 2015). During reclamation, the substrate was not further improved and no other plant species were purposely introduced. The understory of the alder stands could, therefore, be considered as independently developing (Mudrák et al., 2010). The development of the herb understory is faster than at the spontaneous succession, but the understory vegetation cover decreases with site age as the canopy of the alder trees becomes denser.

The sampling design was determined by existent spatial distribution of differently aged areas within the spoil bank. Both the spontaneously developing and the reclaimed chronosequence were studied in four successional stages (*c.* 12, 20, 30, and 50 years old, **Figure 1**). Generally, each combination of management and successional stage were replicated at three sites (one specific combination was studied at four sites and one at two sites, see Supplementary Figure S1). The sites were selected as distant from each other as possible (at least 100 m apart) with a stripe of different vegetation cover or successional stage separating relatively nearby situated replicates.

### Root and Soil Sampling

*Calamagrostis epigejos* (Poaceae), a perennial rhizomatous grass, was selected for the analysis of AMF diversity at the sites. It belongs to the first spoil bank colonizing AMF hosts and is abundant in all successional stages under both managements. At the spontaneously developing chronosequence, it is one of two understory species with the highest cover, which increases with successional age (Frouz et al., 2008). At the reclaimed chronosequence, *C. epigejos* clearly dominates the understory (Mudrák et al., 2010).

Two samplings were carried out, in June (spring) and September (autumn) 2013. At each sampling event, six root systems of *C. epigejos* were randomly collected at each site, totaling in 288 root systems. Each root system was placed into a plastic bag together with the surrounding soil. The root systems with adhering soil were placed separately into plastic bags and transported to the laboratory within 12 h, where they were kept in 4°C until processing (but no more than 2 days). Roots were subsequently washed and healthy looking turgescent roots were cut into pieces. From each root system, one random subsample of 0.1 g fresh weight was frozen in liquid nitrogen and stored at -80°C for DNA isolation. The remaining roots were stained with Trypan blue (Koske and Gemma, 1989) and the intensity of colonization by AMF was measured under a compound microscope at 100× magnification according to Trouvelot et al. (1986) using 30 root segments of 1.5 cm in length.

### Determination of Soil Chemical Properties and Neighboring Plant Communities

Soils collected together with the roots were mixed, homogenized, air-dried, sieved on 2 mm sieve and subjected to soil chemical analyses (**Table 1**). Soil pH and conductivity were measured in deionized H_2_O (1:5 w/v sample/liquid ratio). Total carbon (C_tot_) and nitrogen (N_tot_) concentrations were analyzed on a CHN Carlo Erba EA 1108 analyzer (Italy). Total phosphorus (P_tot_) concentration was measured spectrophotometrically at 889 nm after mineralization in nitric and perchloric acids (GENESYS 10S UV-VIS, Thermo Scientific, USA). Available P (P_avail_) was extracted from the soil samples using the Mehlich 3 method (1:100 w/v sample/extraction agent ratio) and measured spectrophotometrically at 889 nm (GENESYS 10S UV-VIS, Thermo Scientific, USA). Available calcium (Ca_avail_), magnesium (Mg_avail_) and potassium (K_avail_) were extracted using the same method, but with a 1:10 w/v sample/extraction agent ratio, and analyzed using ion chromatography (Dionex ICS-5000 equipped with PDA photodiode array detector, Thermo Scientific, USA).

**Table 1:** Soil characteristics of the spontaneously developing (S) and reclaimed (R) sites of successional stage.

			Successional stage (age in years)			
			
			12	20	30	50
pH_H_2_O_		S	7.60 ± 0.09 a	7.86 ± 0.08 a	7.78 ± 0.03 a	7.22 ± 0.02 b
		R	7.95± 0.02 a	7.84 ± 0.05 a	7.6 ± 0.15 a	6.92 ± 0.17 b
Conductivity	μS cm^-1^	S	217 ± 17 a	184 ± 5 ab	179 ± 8 ab	170 ± 16 b
		R	181 ± 4 a	185 ± 10 a	196 ± 15 a	180 ± 26 a
C_tot_	%	S	6.21 ± 0.14 b	4.76 ± 0.18 c	6.7 ± 0.24 ab	7.39 ± 0.55 a
		R	4.68 ± 0.14 c	6.36 ± 0.57 b	8 ± 0.57 a	8.38 ± 0.41 a
N_tot_	%	S	0.40 ± 0.01 b	0.44 ± 0.02 b	0.49 ± 0.01 a	0.39 ± 0.02 b
		R	0.38 ± 0.02 b	0.41 ± 0.05 b	0.56 ± 0.04 a	0.57 ± 0.04 a
P_tot_	mg kg^-1^	S	760 ± 48 a	978 ± 86 a	785 ± 51 a	832 ± 51 a
		R	814 ± 93 a	814 ± 90 a	796 ± 88 a	798 ± 79 a
P_avail_	mg kg^-1^	S	48.8 ± 3.3 a	28.1 ± 1.9 b	50.0 ± 6.9 a	62.1 ± 4.31 a
		R	38.8 ± 4.34 a	44.8 ± 6.38 a	40.4 ± 5.34 a	31.1 ± 3.95 a
Ca_avail_	mg kg^-1^	S	7770 ± 367 a	8035 ± 330 a	5154 ± 275 b	3751 ± 333 c
		R	6597 ± 425 a	5350 ± 382 b	4856 ± 519 b	3213 ± 231 c
Mg_avail_	mg kg^-1^	S	1251 ± 45 b	1494 ± 32 a	1535 ± 42 a	1148 ± 82 b
		R	1413 ± 26 ab	1466 ± 66 ab	1335 ± 102 b	1569 ± 73 a
K_avail_	mg kg^-1^	S	297 ± 9 b	327 ± 13 b	381 ± 21 a	412 ± 25 a
		R	285 ± 8 b	403 ± 19 a	335 ± 19 b	394 ± 17 a


*Data are means of three replicates ± SE. Different letters indicate; within each parameter; significant differences according to nested ANOVA (site nested within successional stage) followed by Tukey’s HSD test (*P* < 0.05). The determination of the soil parameters is explained in the corresponding section of “Materials and Methods”.*

At the autumn sampling, we recorded all plant species present in a circle of radius of 0.75 m around the sampled *C. epigejos* plants. The neighboring plant communities of each site were then described as frequency of plant species in the six circles (Supplementary Table S1).

### Molecular Analysis

DNA was extracted from the frozen root samples using NucleoSpin Plant II Kit (Macherey-Nagel, Germany) according to the manufacturer’s instructions and eluted in 100 μl double distilled water (ddH_2_O). A 500 to 600 bp long fragment of the nuclear large subunit ribosomal DNA (LSU rDNA) of AMF was amplified in a nested PCR approach using the AMF specific primer mixtures SSUmA/LSUmA (Krüger et al., 2009) in the first step and LSUmB (Krüger et al., 2009) in combination with a modified 250f primer (Sýkorová et al., 2012; 5′-AGTTGTTTGGGAWTGCAGCT-′3) in the second step. Both primers were tagged with Molecular Identifiers (MID) suggested by Roche (2009) (454 Sequencing Technical Bulletin No. 005-2009). All DNA extracts were diluted 1:100 with ddH_2_O. Five μl of diluted DNA extract were used as template in the first PCR, 0.6 μl of the first PCR was used as template in the nested PCR. The PCRs were conducted with Taq Pfu DNA Polymerase (Thermo Scientific) with final concentrations of 1x Pfu buffer, 2 mM MgSO_4_, 200 μM of each dNTP and 0.5 μM of each primer in a total volume of 20 or 25 μl. The PCR conditions were as follows: initial denaturation at 95°C for 5 min, followed by 40 cycles with 95°C for 5 min, 58°C/59°C (first PCR/nested PCR) for 45 s and 72°C for 2 min, concluded by a final elongation at 72°C for 10 min. PCR products were visualized on a 1% agarose gel. Successfully amplified samples were loaded on a 1% agarose gel and bands of the expected size excised and processed with the Zymoclean Gel DNA Recovery Kit (Zymo Research, USA). The DNA concentration in the eluates was measured using a Qubit 2.0 Fluorometer with the HS kit.

Products from the six root samples per sampling time and site were equimolarly pooled together, resulting into 48 pooled samples, one per each sampling time and site. The pools were sent either to GATC Biotech (Konstanz, Germany, first pool) or to Macrogen (Seoul, South Korea, second – fourth pool) for 454 sequencing (GS FLX platform, Roche). Raw sequence data and associated metadata are available from the PlutoF repository^1^.

### Bioinformatical Analysis

Sequence reads were demultiplexed according to the samples-specific barcodes, quality filtered (minimum quality score 20, primers kept, maximum 2 primer mismatches), and trimmed to a minimum length of 300 bp, using QIIME (Caporaso et al., 2010) command –split_libraries.py. The complete dataset was checked for chimeric sequences and removed by UCLUST (Edgar, 2010), and Operational Taxonomic Units (OTUs) were *de novo* clustered at 97% similarity threshold by USEARCH, using the SEED workbench (Větrovský and Baldrian, 2013). Global singletons were removed from the data set. All reads of each primary cluster were merged to consensus sequences, using MAFFT’s iterative refinement method (L-INS-i). These consensus representative sequences were clustered a second time at 97% as described in Kohout et al. (2015) and Krüger et al. (2015), and we refer to these secondary clusters as ‘OTUs.’ Affiliation to AMF was checked by BLAST against the public databases (DDBJ/EMBL/GeneBank). According to the closest BLAST non-target sequences were excluded from further analyses and the remaining sequences were manually aligned against a backbone database published in Krüger et al. (2012). The maximum-likelihood phylogenetic backbone tree was calculated using RAxML (Stamatakis, 2006) over the Cipres web-portal (GTRGAMMA, 1000 bootstraps), and for assignment to phylogenetic taxa the Evolutionary Placement Algorithm (EPA) for short sequence reads (Berger et al., 2011) was used implemented in QIIME (command –insert_seqs_into_tree.py). These, subsequently called ‘AMF taxa,’ were labeled according to their taxonomical clustering in the calculated phylogenetic EPA tree, as it provides an accurate phylogenetic placement of short reads (Senés-Guerrero and Schüßler, 2015).

### Statistical Analysis

#### Data Transformation and Calculation of Distance Matrices

Only samples with more than 72 AMF reads were considered for the statistical analyses, resulting into a set of 37 samples (Supplementary Table S2). Prior to the statistical analyses, residuals of AMF OTU richness (number of AMF OTUs per sample) or residuals of AMF taxon richness (number of AMF phylogenetically defined taxa per sample) were calculated using the *resid* function of linear correlation between the sequence numbers and AMF OTU/taxon richness. The residuals were subsequently used in statistical analyses to overcome the potential effect of different sequencing depth between samples.

Prior to the subsequent analyses, fungal communities were standardized using Hellinger transformation. Bray–Curtis dissimilarity measure was used to construct fungal community dissimilarity matrix. Euclidean dissimilarity measure was used to construct soil chemistry and site age dissimilarity matrices. For categorical environmental variables [sampling time (June vs. September) and management regime (unreclaimed vs. reclaimed)], the Gower’s dissimilarity metric was calculated in the ‘cluster’ package of R (R Core Development Team, 2008). We determined plant species, whose occurrence was significantly related with AMF community composition using *ordistep* function in ‘vegan’ package of R. Only plant species recorded from at least three sites were used for the analysis. Plant species showing significant relationship with AMF community composition were used in subsequent model selection analysis, variation partitioning and linear regression with Hellinger transformed sequence data of AMF occurrences.

#### General Least Squares Modeling

A general least squares (GLS) model was built to identify the main predictors of mycorrhizal root colonization and AMF OTU/taxon richness, based on the following parameters: site age, management, number of accompanying plant species, plant community matrix with selected significant plant species (as described above) and soil characteristics (pH, soil conductivity, C_tot_, N_tot_ P_tot_, C : N, P_avail_, Ca_avail_, K_avail_, and Mg_avail_), using the routine of the ‘nlme’ package of R (Pinheiro et al., 2008). Multicollinearity between predictor variables was checked by calculating the variance inflation factor (VIF). Variables with VIF > 5 were excluded from the model selection. Collinearity with other environmental characteristics was found only in the case of N_tot_, which was therefore also removed from the model. The best model was selected according to the corrected Akaike information criterion (AICc). Robustness of the best model was further evaluated by averaging models that fell into the 95% AICc confidence set. Beta coefficients (slopes) of individual models were weighted according to their Akaike weight across all models and evaluated as the mean ± 95% confidence intervals. Zero values were conservatively used for non-significant variables in individual models. Variables were considered significant when confidence intervals excluded zero values.

#### Analysis of AMF and Plant Composition Variation

We also analyzed the response of AMF and plant composition variation to site age. Composition variation was defined following Anderson et al. (2011) as variation beta diversity, which is the variance calculated for the different samples per site age and management regime. For statistical comparison, the composition variation was calculated using the functions *betadisper* and *permutest.betadisper* in the ‘vegan’ package of R (Oksanen et al., 2012).

#### Multivariate Analysis

PERMANOVA of the Adonis routine of the ‘vegan’ package was used to address the relative importance of edaphic characteristics, spatial distance among the sites, site age, management, sampling time and plant community composition (represented by the selected significant plant species) for AMF community succession. The AMF OTU abundances or AMF taxon abundances across samples were standardized using Hellinger transformation, which allows the use of linear-based ordination also for non-linear data (Legendre and Gallagher, 2001). The effect of spatial distance was taken into account by reducing the Euclidean distance matrix into spatial Principal Coordinates of Neighbor Matrices (PCNM) that accounted for spatial autocorrelation at different scales (Borcard and Legendre, 2002). Adonis tests the significance of discrete and continuous factors based on permutations. Adjusted *R*^2^ were calculated based on the Adonis results in order to check for model quality. Bray–Curtis distances of the plant and AMF communities were used for non-metric multidimensional scaling (NMDS) analyses in the ‘ecodist’ (Goslee and Urban, 2007). Confidence ellipses (95% confidence interval) for the successional stages were calculated with the function *ordiellipse* in ‘vegan’ package. Indicator AMF taxa for significant environmental characteristics were determined using the *indVal* function of the ‘labdsv’ package of R (Roberts, 2014). The *varpart* function in the ‘vegan’ package was used to partition the variance of AMF community dissimilarity into contribution by edaphic factors (only soil characteristics identified by step wise selection as significant predictors of AMF communities composition were used), plant community composition, successional stage and spatial variables. Significance of AdjR^2^ values of variance partitioning was tested using redundancy analysis (RDA).

To test potential effect of unequal number of sequences per sample, all samples with higher number of sequences than 500 were sub-sampled to the same level (500 sequences per sample). Samples with less than 500 sequences were not changed. This sub-sampling depth represents approximately the lower quartile of the dataset. This strategy was previously proven by de Cárcer et al. (2011) to sufficiently overcome problems with unequal sampling intensity. Sub-sampled dataset was subsequently used for calculating PERMANOVA. These results were compared with the results obtained from original dataset.

## Results

### Phylogenetic Classification of AMF

In total, 176,057 short sequence reads of the LSU rDNA were obtained after the second clustering at the 97% sequence identity threshold. BLAST searches assigned 81.5% of all reads to Glomeromycota and 18.5% to other organisms (e.g., plants, Asco- and Basidiomycota). The 143,337 target sequence reads corresponded to 363 AMF OTUs (Supplementary Table S3), which were assigned to 40 phylogenetically supported AMF taxa (Supplementary Figure S2 and Table S4). Most OTUs were affiliated with AMF taxa assigned to the genus *Dominikia* (155 OTUs), followed by *Rhizophagus* (127 OTUs) and *Claroideoglomus* (47 OTUs). Most sequence reads originated from members of the families Glomeraceae (83.5%), Claroideoglomeraceae (15.3%), and Diversisporaceae (1.1%).

### AMF Taxon Richness and Composition Variation

Arbuscular mycorrhizal fungal richness varied from one to 24 taxa per sample, with an average of 9 taxa (Supplementary Table S4). The best explanatory model (AICc = 201.04) for AMF richness included sampling time (*t* = -2.49, *P* = 0.018), pH (*t* = 2.17, *P* = 0.038) and soil conductivity (*t* = 1.61, *P* = 0.12). The averaged model was built on 212 models (AICc ranged between 201.04 and 327.29). Based on the 95% confidence interval, only sampling time (June vs. September) had a significant effect on AMF taxon richness among the studied variables, with a higher richness found in June than September (Supplementary Figure S3).

**FIGURE 2:**
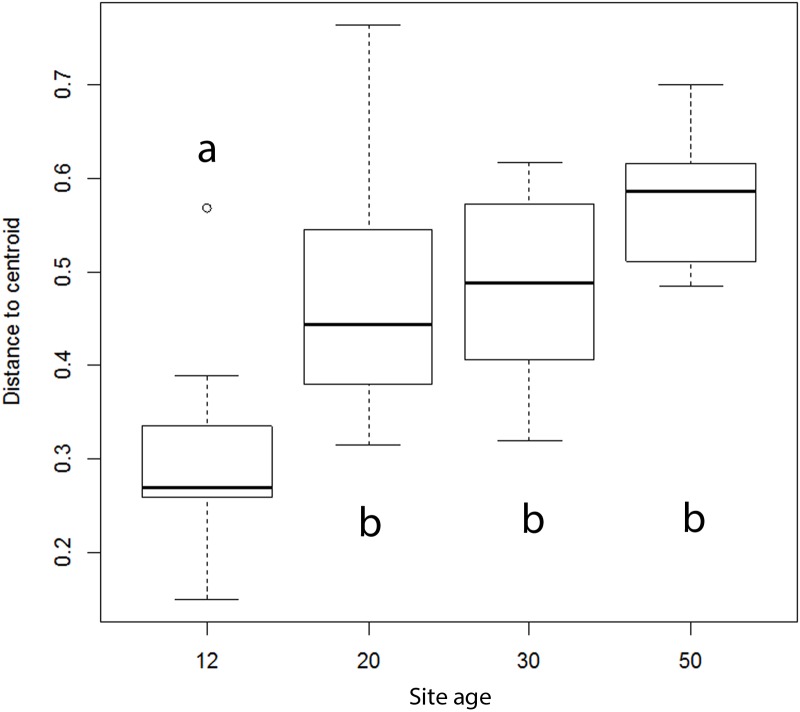
**Effect of the successional stage on composition variation of arbuscular mycorrhizal fungal communities, as indicated by AMF species variation.** Data are pooled for the two chronosequences (spontaneously developing and reclaimed) and for the two samplings (in June and September), which resulted in 13 samples for 12-year-old, eight samples for 20-year-old, nine samples for 30-year-old and five samples for 50-year-old site. Bold line represents median and the bottom and top of the box represent lower and upper quartiles. Different letters indicate significant differences among the successional stages at *P* < 0.05.

According to a permutation test, AMF composition variation showed a significantly positive correlation with successional age (df = 3, *F* = 11.25, *P* < 0.001, **Figure 2**). The youngest 12-year-old sites showed significantly lower variation in AMF composition than older successional sites. Mean levels of composition variation of the intermediate sites (20 and 30 years) was intermediate between the youngest and oldest sites, but because of large variation, differences were not significant among the other successional stages. *Dominikia* sp. 1 was the most abundant (based on sequence numbers) in eight out of 13 samples from the earliest successional stage (*P* < 0.001, *R*^2^ = 0.47, *F* = 31.16, Supplementary Figure S4 and Table S4).

### AMF Community Composition

**FIGURE 3:**
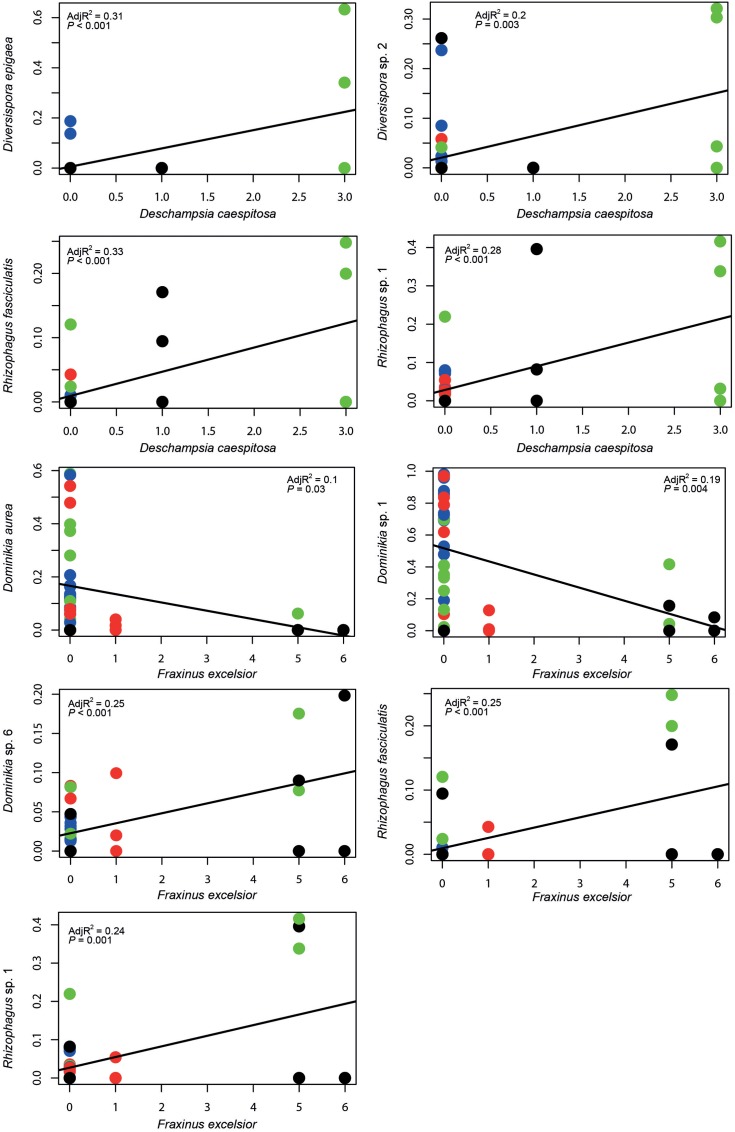
**Plots of AMF fungal taxa occurrences (based on Hellinger transformed sequence data) against neighboring plants incidences.** Two out of the five plant species, identified by the forward selection as significant determinants of AMF community composition, are presented, because of their higher abundance. Remaining three plant species occurred on less than four sites only and therefore they were not included in the regression analysis. Color represents different chronosequence sites: blue, 12-years-old site; red, 20-years-old site; green, 30-years-old site and black, 50-years-old site. The line represents the best fitting linear model.

Prior the PERMANOVA analysis, we identified five plant species (**Table 2**), whose occurrence significantly affected AMF communities. Altogether, these selected plant species explained 24% of the variability in AMF community composition. Species matrix of these five plants was used as plant community composition factor in PERMANOVA and variation partitioning analysis. Besides that, AMF fungal taxa with significant correlation to the selected plant species were determined (**Figure 3**).

**Table 2:** Plant species showing significant relationship with AMF community composition.

	*F*_statistics_	Adj. *R*^2^	*P*-value
*Fraxinus excelsior*	3.43	0.063	0.004
*Chamerion angustifolium*	2.91	0.048	0.004
*Pastinaca sativa*	2.73	0.044	0.024
*Acer pseudoplatanus*	2.73	0.042	0.014
*Deschampsia caespitosa*	2.43	0.034	0.018


Stepwise selection of factors identified spatial distance (represented by two significant PCNM vectors), site age, plant community composition, soil K_avail_, Ca_avail_, and Mg_avail_ concentrations and soil conductivity as the best explanatory factors for AMF community composition. However, only spatial distance (*F* model = 5.8, *P* < 0.001) and plant community composition (*F* model = 2.89, *P* < 0.001) were significant in the final model and explained 15.4 and 12.8% of total variance (based on the AdjR^2^ values).

**FIGURE 4:**
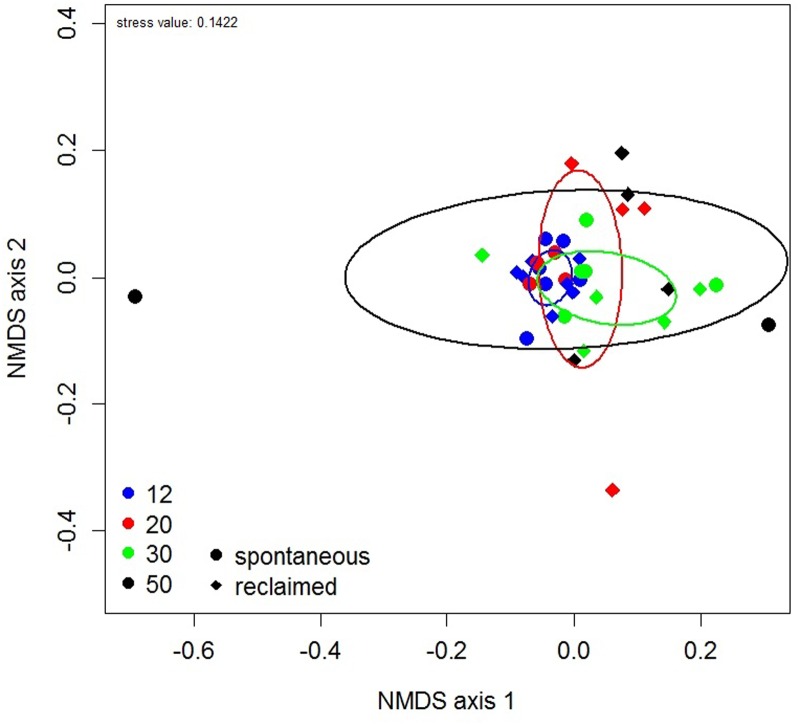
**Non-metric multidimensional scaling (NMDS) plot of community composition of phylogenetically defined arbuscular mycorrhizal fungal taxa.** Each symbol represents one pooled root sample. Ellipses represent ordination confidence intervals (95%). Site age is indicated by color and management regime by shape of the symbol.

The NMDS ordination revealed increasing dispersion in AMF community composition with successional age (**Figure 4**), which was congruent with the observed differences in AMF composition variation as described above. Samples of the intermediate stages (20 and 30 years) showed wider scattering in the ordination plot then those of the earliest stage, but still less than those of the oldest one. In accordance with PERMANOVA, the AMF community composition did not differ among the successional stages as indicated by overlapping confidence ellipses in the NMDS plot.

**FIGURE 5:**
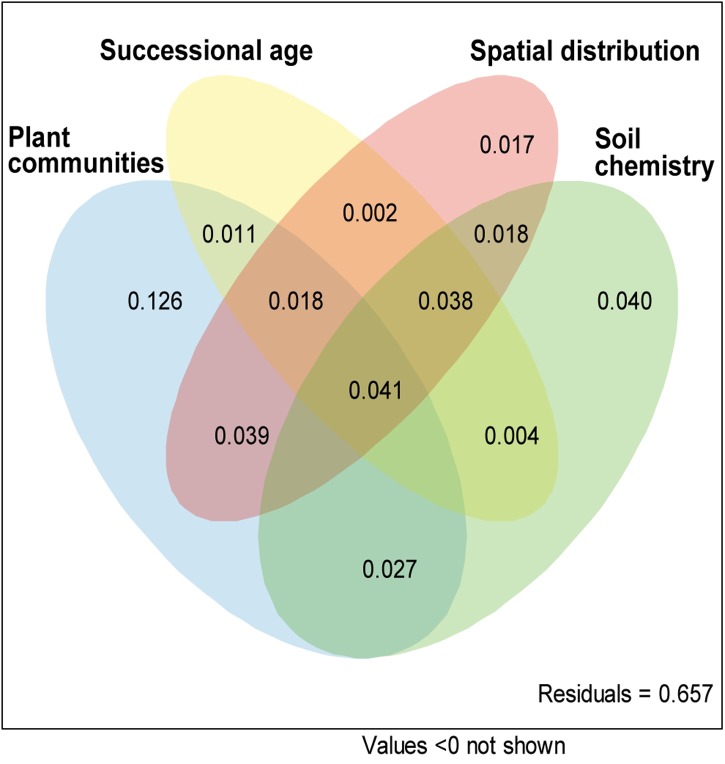
**Pure and shared effects of plant communities, site age, spatial distribution and soil chemistry on AMF community as derived from variation partitioning analysis.** Numbers indicate the proportion of explained variation.

The variation partitioning analysis revealed plant community composition (AdjR^2^ = 0.338, *P* < 0.001), site age (AdjR^2^ = 0.128, *P* < 0.001), spatial distance (AdjR^2^ = 0.193, *P* < 0.001) and soil chemistry (AdjR^2^ = 0.233, *P* = 0.006) as significant factors in the AMF community composition. However, most of the explained variability was shared among different explanatory variables, only plant community showed pure significant covariation with AMF communities (**Figure 5**, *P* < 0.001) and explained 12.6% of total variance.

Compared with phylogenetically defined AMF taxa (40), OTU delimitation by 97% sequence identity rendered an order of magnitude more taxa, namely 363 OTUs (Supplementary Table S3). However, OTU-based PERMANOVA and NMDS analysis rendered the same results as when based on the phytotaxa (Supplementary Figure S5). Similarly, there were no differences of PERMANOVA and AMF composition variation results between sub-sampled (Supplementary Table S5) and original datasets (results presented in Supporting Information).

### Plant Diversity

Plant richness varied from eight to 30 species per sample, with an average of 13 taxa (Supplementary Table S1). The best average model (AICc = 213.36) for the effect of the environmental variables on plant species richness included site age (*t* = 3.06, *P* = 0.004) and pH (*t* = 2.12, *P* = 0.042), and was built on 318 models (AICc ranged between 213.36 and 364.01). Based on the 95% confidence intervals of beta coefficients, only site age had a consistently significant positive effect on plant species richness among all considered explanatory variables. According to a permutation test, plant composition variation showed no significant correlation with site age (Supplementary Figure S6).

**FIGURE 6:**
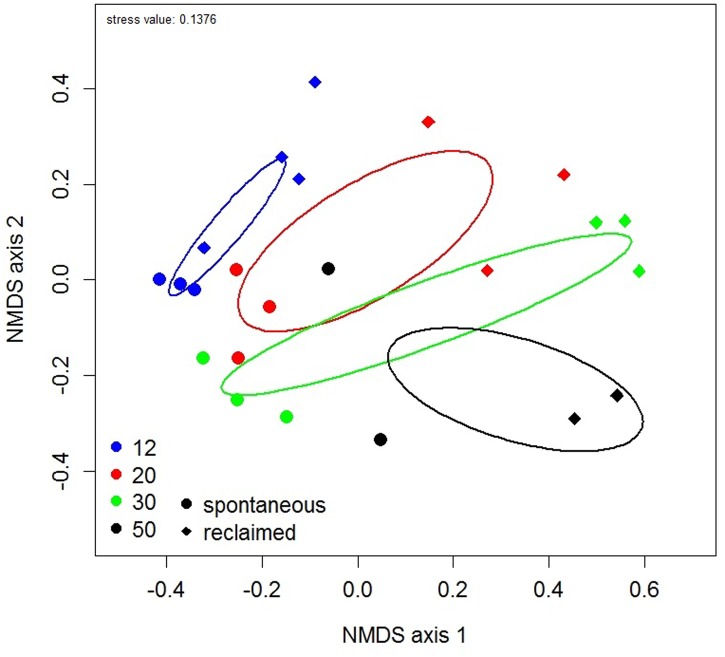
**Non-metric multidimensional scaling (NMDS) plot of the composition of plant communities.** Each symbol represents the plant community of one studied site. Ellipses represent ordination confidence intervals (95%). Site age is indicated by color and management regime by shape of the symbol.

Stepwise selection of predictors identified spatial distribution (characterized with seven significant PCNM vectors), management, site age, soil Mg_avail_ and Ca_avail_, soil conductivity, C: N and C_tot_ as the best explanatory factors for the composition of the whole plant community. However, only spatial distance (*F* model = 5.98, AdjR^2^ = 0.362, *P* < 0.001), management (*F* model = 12.99, AdjR^2^ = 0.126, *P* < 0.001) and site age (*F* model = 3.17, AdjR^2^ = 0.01, *P* = 0.007) were significant in the final model. Congruently, NMDS ordination (**Figure 6**) showed significant effects of management and of site age on plant community composition.

### Mycorrhizal Root Colonization

**FIGURE 7:**
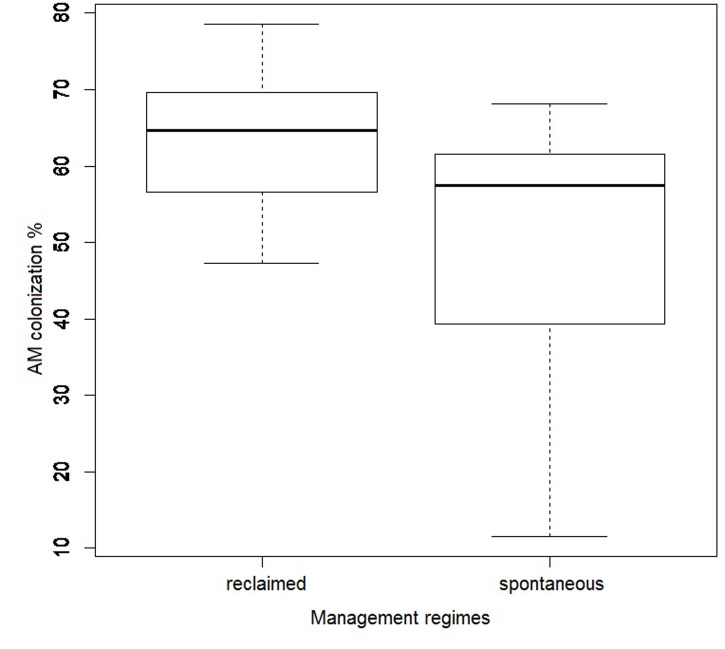
**Effect of management regime (spontaneous vegetation development vs. reclaimed by Alnus plantations) on root colonization by arbuscular mycorrhizal fungi.** Bold line represents median and the bottom and top of the box represent lower and upper quartiles.

All sampled *C. epigejos* plants had their roots colonized by AMF, with mycorrhizal colonization ranging from 12 to 79%, with an average of 57%. According to AIC model selection (AICc = 290.09), mycorrhizal root colonization was best explained by management regime (spontaneous vs. reclaimed, *t* = -3.96, *P* < 0.001) and C_tot_ in soil (*t* = -3.04, *P* = 0.005). The averaged model was built on 149 models (AICc ranged between 290.09 and 352.65). Only management had a consistently significant effect on mycorrhizal colonization considering the 95% confidence intervals of beta coefficients, with higher values for samples from the reclaimed sites (**Figure 7**).

## Discussion

This study focused on soil chemistry and neighboring plants as possible factors affecting AMF community development in the roots of one constant host plant during primary succession. It has not revealed any correlation between the measured soil characteristics and AMF community composition despite changes in soil chemistry over the studied chronosequences. However, the composition of AMF community covaried with the neighboring plant community composition, which points at a more important role of biotic rather than abiotic factors in AMF community assembly during primary succession. Furthermore, our results show that AMF composition variation rather than richness is positively correlated with successional stage.

It is well established that plant species richness generally increases during primary vegetation succession (Walker and Del Moral, 2003), which was also the case over the studied chronosequences (12–50 years). Contrary to our expectation, however, the higher diversity of the neighboring plant community did not increase the AMF species richness in the roots of *C. epigejos*. Although our data are based on a single host plant only, the results confirm a weak, or even negative correlation between AMF and plant species richness during primary succession (Johnson et al., 1991; Sikes et al., 2012; Zangaro et al., 2012; Martínez-García et al., 2015), in contrast to the situation encountered in stable ecosystems (Hiiesalu et al., 2014). The contrasting patterns in the dynamics of plant and AMF richness in ecosystem development may be on one hand explained by the contrasting levels of inter and intra-specific variability between plants and AMF taxa (Bruns and Taylor, 2016; Öpik et al., 2016). On the other hand, the explanation may lie in differential speeds of dispersal in the two groups of organisms. As previously shown by Davison et al. (2015), AMF seem to lack dispersal limitation, at least on larger spatial and temporal scales. We therefore hypothesize that AMF dispersal to and establishment at new sites is much faster and more efficient than that of plants.

While AMF taxon richness was unaffected by successional stage, AMF composition variation was positively correlated with the age of the successional stages. This relationship contradicts our expectation that the AMF communities of *C. epigejos* become established by random filtering of the local species pool. Most fungal communities from the earliest successional stage were dominated by the same AMF species, i.e., shared *Dominika* sp. 1 as the most abundant AMF taxon. With increasing site age, however, the most abundant AMF taxon became more variable. This could originate from two different mechanisms. First, early-stage AMF community assembly may have been deterministic, the early-stage conditions favoring population growth of (one) compatible species, while stochastic factors dominated in later stages with conditions favorable to a broader spectrum of species. Such a scenario is supported by previous studies in mature ecosystems that found evidence for a prevalence of stochastic processes in the structuring of AMF communities and other soil microbial communities (Powell et al., 2009; Bahram et al., 2016). Alternatively, increasing habitat diversity with ecosystem age (Walker and Del Moral, 2003) may lead to increased patchiness in the AMF communities of mature ecosystems. To further disentangle changes in the relative contribution of deterministic and stochastic processes to AMF community assembly in primary succession; more detailed sampling is need. Particularly, a spatially explicit design for each successional stage along the chronosequences would provide more detailed information.

Vegetation development in primary succession is inseparably related to pedogenesis. Soil chemistry was previously described as a key factor influencing AMF community assembly (Lekberg et al., 2007; Fitzsimons et al., 2008). However, neither a single soil chemical parameter (PERMANOVA) nor soil chemistry itself (variation partitioning) showed direct significant effect on the AMF communities in this study despite changes in soil chemistry during the ecosystem development, such as increase of soil carbon or decrease of available calcium and soil pH, which might also influence P availability in the soil. Besides, soil chemical parameters, soil physical properties, such as soil texture and structure might also change during the ecosystem development. Herrmann et al. (2016) previously described a significant effect of soil physical properties on the composition of AMF communities. Further clarification concerning the effect of soil physical properties on development of AMF communities during primary succession is needed.

On the contrary, the composition of AMF communities in the roots of *C. epigejos* was significantly correlated with composition of neighboring plant species. Interestingly, none of these five plant species occurred at the earliest successional stage, which indicates increasing covariation between AMF and plant communities during primary succession. Covariation between plant and AMF communities has been reported repeatedly (Fitzsimons et al., 2008; Hausmann and Hawkes, 2009; van de Voorde et al., 2010; García de León et al., 2016). However, this study for the first time shows that variation in root-colonizing AMF communities is more connected to neighboring plant communities than to soil chemistry during primary succession. This expands the conclusion of Martínez-García et al. (2015) of host plant identity as a major driver of AMF community composition during primary succession, broadening host plant identity to plant community. Altogether, both studies support the Passenger/Driver hypothesis (Hart et al., 2001) rather than habitat filtering hypothesis (Zobel and Öpik, 2014) of AMF community assembly during primary succession.

Most AMF communities of the earliest successional stage were dominated by *Dominikia* sp. 1 (sister clade to *Rhizophagus*), whose abundance gradually diminished along the chronosequences. Our finding is in agreement with Chagnon et al.’s (2012) trait-based framework for AMF ecology. These authors proposed that life history traits of members of Glomeraceae are consistent with a ruderal life style. Species of the Glomeraceae family were, indeed, described as early colonizers on a newly exposed artificial island (Nielsen et al., 2016). However, other Glomeraceae species were found to be abundant in the later successional stages, and we still do not know the specific life history traits (e.g., dispersion efficiency) that have enabled *Dominikia* sp. 1 and the other pioneering AMF species to reach dominance at early successional stages.

Besides the plant species ability to grow in ruderal environment, dispersal limitation is the major force structuring plant communities during primary succession (Lichter, 2000). Much less is, however, known about the role of dispersal limitation in AMF communities (Davison et al., 2015). In our study, we observed that roots of *C. epigejos* plants harbored almost all the detected AMF species already at the earliest successional stage. This observation suggests that AMF dispersal was less limited in the present study system than that of Nielsen et al. (2016), who found that the AMF community from the earliest successional stage (also 12 years old) was a non-random subset of the communities found in the later successional stage. A potential explanation for the contradictory results may be different effectiveness of vectors of AMF propagule dispersal in the two systems. While Nielsen’s study focused on an artificial island with very restricted human access, our study was conducted on a spoil heap, which was separated from the surrounding ecosystems by more permeable barriers and exposed to relatively higher human activity (connected to ongoing depositing of spoil in other parts of the spoil heap). We therefore assume that in Nielsen’s study, the vectors of AMF dispersal were just birds and wind, which are relatively insufficient vectors of AMF dispersal (Egan et al., 2014). In contrast, movement of mammals and human activity may have contributed to AMF dispersal in the present study system (Mangan and Adler, 2002; Rosendahl et al., 2009) and by facilitating AMF dispersal, these factors also may have accelerated AMF succession. This indirect comparison therefore highlights the importance of specific site settings, especially the interconnectivity with surrounding biotopes.

Interestingly, we did not find any effect of the spoil heap reclamation either on AMF species richness or community composition in the roots of *C. epigejos*. Previous studies focusing on AMF species compositional changes after reclamation did not make comparisons with a natural successional chronosequence (e.g., de Souza et al., 2013; Zhang and Chu, 2013; da Silva et al., 2015). Our expectation to find different AMF communities in the reclaimed and spontaneous succession chronosequence was therefore based merely on the intuitive assumption that reclamation fundamentally affects site conditions. However, it should be mentioned in this context that overall root colonization level of *C. epigejos* was higher in the reclaimed than in the spontaneous succession chronosequence. As also indicated by the model (significant negative relationship of root colonization with C_tot_), this may have been due to accumulation of an organic layer on soils of the later stages of the spontaneous succession chronosequence. As previously reported (Piotrowski et al., 2008; Becklin et al., 2012b), litter of Salicaceae may inhibit the development of AMF root colonization. Thus, our study is the first comprehensive comparison of a reclaimed and a spontaneous succession chronosequence. Although reclamation significantly influenced plant community composition and root colonization by AMF, it did not lead to different AMF communities. It seems that *Alnus glutinosa* planting did not override the effect of site age on the understory plant communities in a way that would be affecting AMF community assembly. The situation may be different in plantations of other trees with more pronounced effect on the understory vegetation (Mudrák et al., 2010).

## Conclusion

Our results support the suggestion of Zobel and Öpik (2014) that biotic host plant–fungus interactions are more important factors for AMF succession during early ecosystem development than abiotic habitat conditions. In this context; it is paradoxical that spoil bank reclamation by tree plantations had no effect on AMF communities and suggests that among host-related factors; only some are linked with AMF community assembly. Their identification in future studies will improve our understanding of AMF succession and possibly also contribute to discerning “Driver” from “Passenger” effects.

## Author Contributions

CK, PK, MJ, DP, JF, and JR planned and designed the research. CK, MJ, DP, and JR conducted fieldwork. CK, PK, MJ, DP, and JR analyzed data. CK, PK, MJ, DP, and JR wrote the manuscript.

## Conflict of Interest Statement

The authors declare that the research was conducted in the absence of any commercial or financial relationships that could be construed as a potential conflict of interest.

## Footnotes

1 https://plutof.ut.ee/#/filerepository/view/805328
