# Supplementary material for: Plant Communities Rather than Soil Properties Structure Arbuscular Mycorrhizal Fungal Communities along Primary Succession on a Mine Spoil
Source: Front Microbiol. 2017 Apr 20;8:719. doi: 10.3389/fmicb.2017.00719 (PMC5397529; doi:10.3389/fmicb.2017.00719)
Supplement: Supplementary file 2 [file Data_Sheet_2.DOCX]

## *Frontiers in Microbiology* Supporting Information

Article title: Factors driving development of arbuscular mycorrhizal fungal communities during primary succession.

Authors: Claudia Krüger, Petr Kohout, Martina Janoušková, David Püschel, Jan Frouz, Jana Rydlová

**Results**

Results calculated based on the sub-sampled dataset:

*AMF beta diversity*

According to the permutation test, variation beta diversity of AMF communities showed significant positive correlation with site age (d.f. = 3; F = 11.15; P < 0.001). The 12-year-old sites had significantly lower variation beta diversity as compared to the other sites.

*AMF communities composition*

Priory the PERMANOVA analysis, we identified five plant species (Table S6), which occurrence significantly affected AMF communities. The selected plant species were exactly the same as those which were identified based on non-subsampled dataset.

Stepwise selection of factors identified geographical distance (represented by a single significant PCNM vector), plant community composition, soil K_avail_, Ca_avail_ and Mg_avail_ concentrations and soil conductivity as the best explanatory factors for AMF community composition. However, only geographical distance (F model = 15.75; *P* < 0.001) and plant community composition (F model = 2.24; *P* < 0.001) were significant in the final model and explained 22.6 and 7 % of the total variance (based on the AdjR^2^ values).

The following Supporting Information is available for this article:

**Fig. S1** Overview of sampled sites at the studied spoil bank.

**Fig. S2** Maximum-likelihood phylogenetic tree of the backbone database **(a)** and truncated maximum likelihood tree delimiting the arbuscular mycorrhizal fungal taxa **(b).**

**Fig. S3** Effect of sampling time on the taxon richness of arbuscular mycorrhizal fungi.

**Fig. S4** Relative abundance (based on Hellinger transformed data) of *Dominikia* sp. 1 regressed against the age of the successional stages (F = 31.16; R^2^ = 0.47; *P* < 0.001).

**Fig. S5** Non-metric multidimensional scaling (NMDS) plot of arbuscular mycorrhizal fungal community composition in roots based on operational taxonomic units.

**Fig. S6** No effect of successional stage on composition turnover of plant communities. Data are pooled for the two chronosequences (spontaneously developing and reclaimed).

**Fig. S1** Overview of sampled sites of spontaneous (dark) and restoration (light) chronosequence at the studied spoil bank, with relative size, position and succession stage (12, 20, 30 and 50 years) of each site displayed. As the fifty years old sites are remote from the other sites, they are shown separately in rectangles and their distances and directions are indicated.


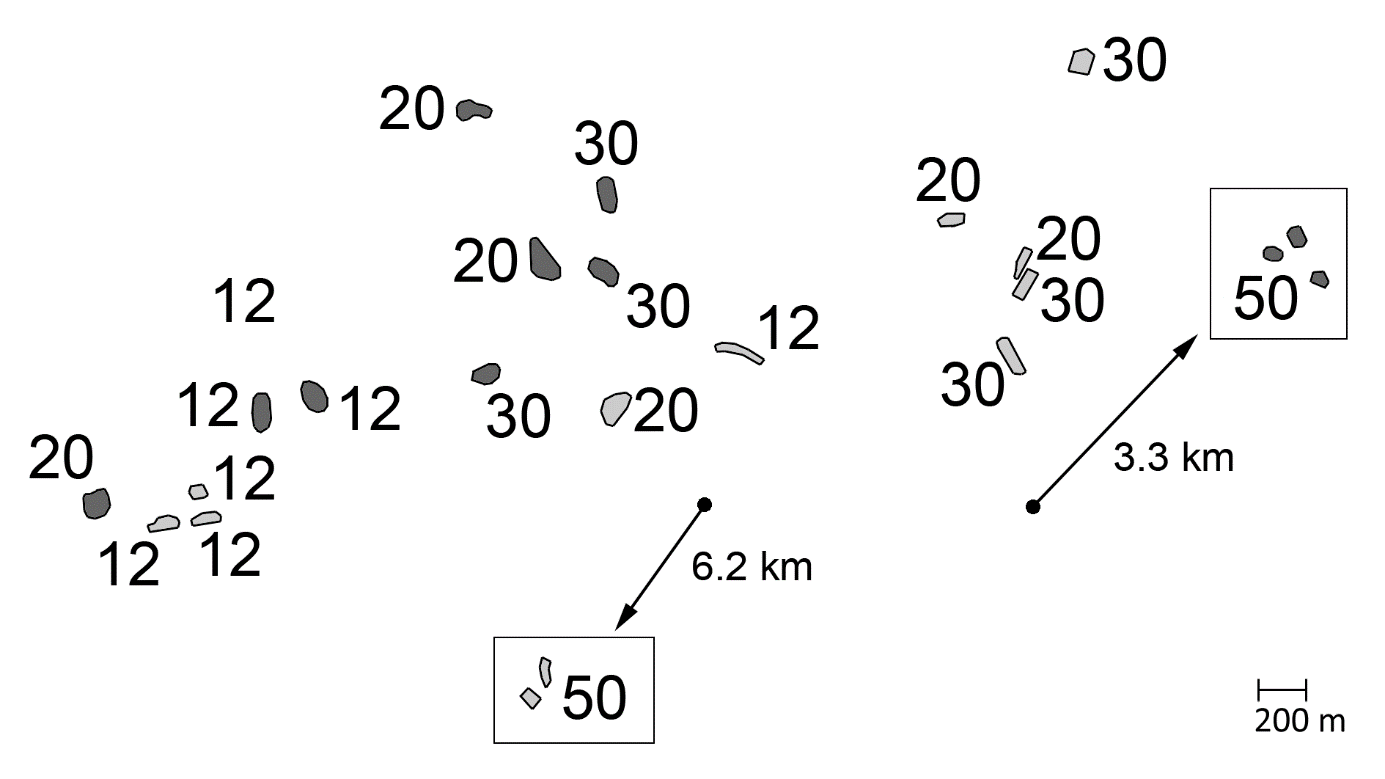


**Fig. S2** Maximum-likelihood phylogenetic tree (after Krüger et al. 2012) **(a)** used as backbone for putative taxonomic placement of representative sequences of AMF OTUs. Main branches with bootstrap support below 60 were collapsed to polytomies. Sequences marked in green correspond to consensus sequences. Grey boxes indicate different phylogenetic clades of arbuscular mycorrhizal fungal (AMF). Outgroup: Asco- and Basidiomycota (AFTOL-IDs 0468,0674,0668,1199). Truncated maximum likelihood tree delimiting the AMF taxa of the study as given out by the EPA tool **(b)**. AMF taxa are marked in blue and labelled according to the corresponding taxonomical positions, the number of operational taxonomic units included in each taxon is given in brackets. Branches marked with / were shortened by 75 %, // shortened by 90 %.

Provided in separate file Fig_S2.pdf


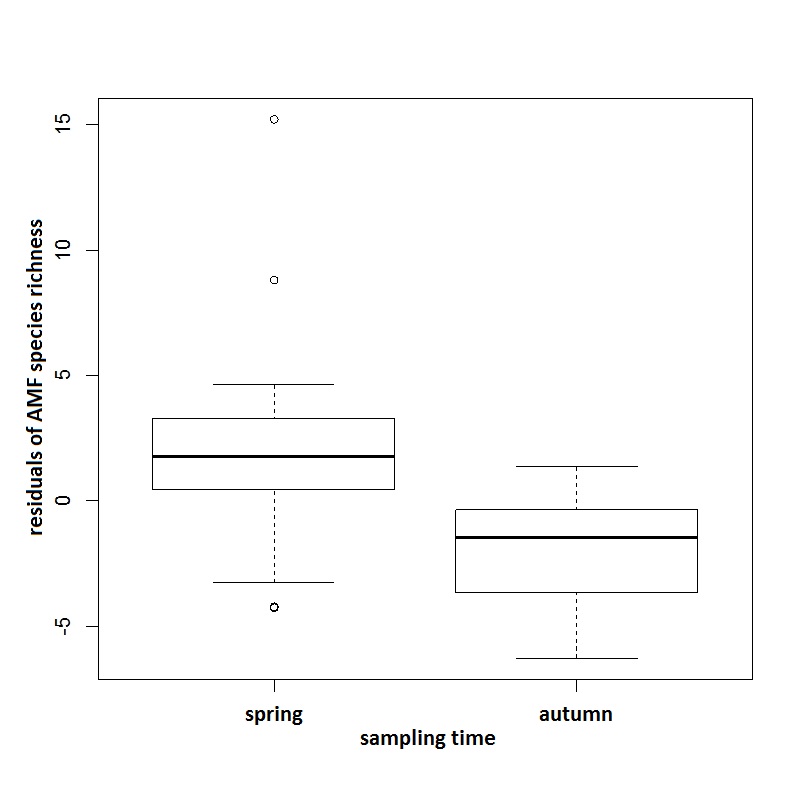


**Fig. S3** Statistically significant effect of sampling time on taxon richness of arbuscular mycorrhizal fungi (t = -2.49; *P* = 0.018).


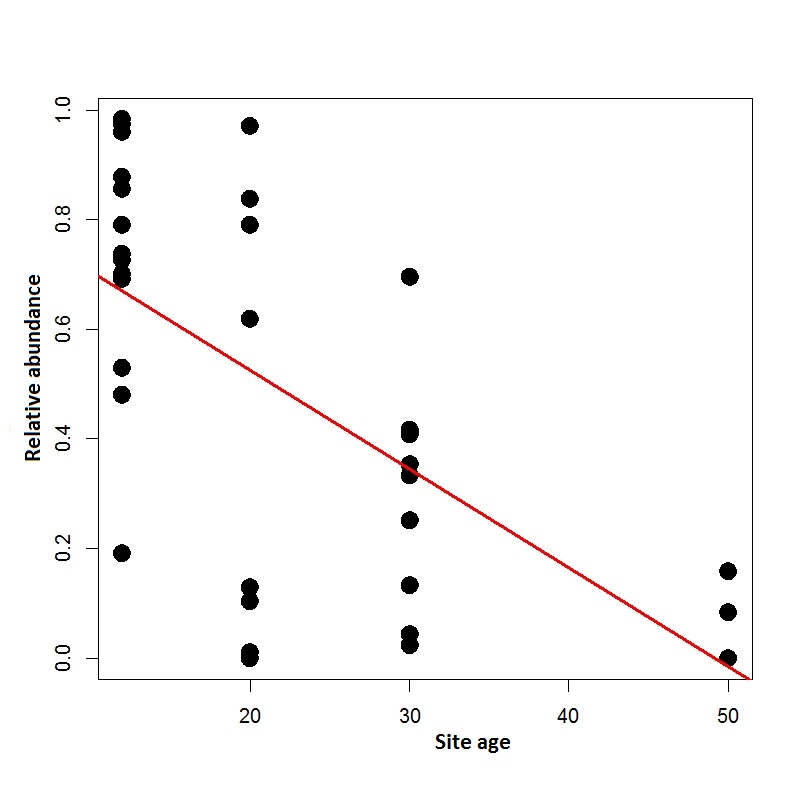


**Fig. S4** Relative abundance (based on Hellinger transformed data) of *Dominikia* sp. 1 regressed against the age of the successional stages (F = 31.16; R^2^ = 0.47; *P* < 0.001).


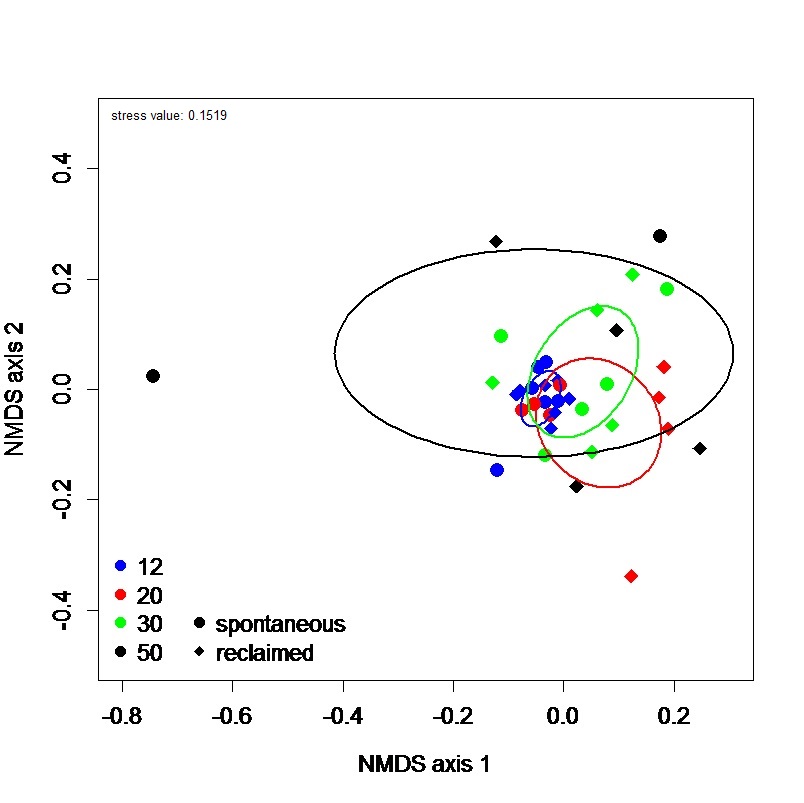


**Fig. S5** Non-metric multidimensional scaling (NMDS) plot of arbuscular mycorrhizal fungal community composition in roots based on operational taxonomic units.. Each symbol represents one pooled root sample. Ellipses represent ordination confidence intervals (95 %). Site age is indicated by color and management regime by shape of the symbol.

**
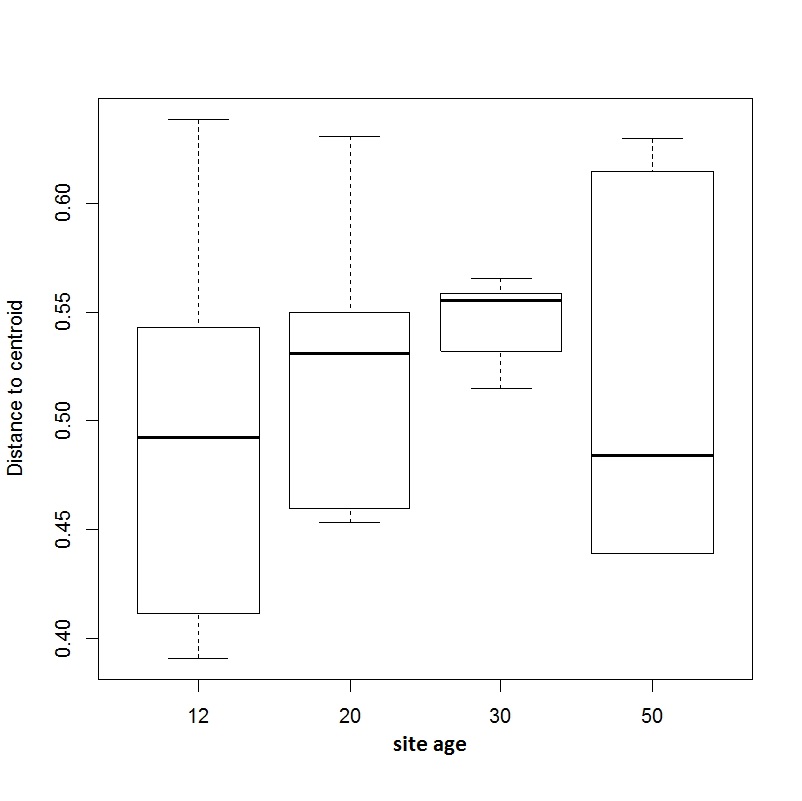
**

**Fig. S6** No effect of site age on variation beta diversity of plant communities. Data are pooled for the two chronosequences (spontaneously developing and reclaimed). Bold line represents median and the bottom and top of the box represent lower and upper quartiles.

**Table S1** Community matrix of plant species. Counts of the plant species in the six plots evaluated per site.

Provided in separate file Table_S1_S3_S4_S5.xls, Sheet Table_S1

**Table S2** Number of pooled AMF community amplicons of *Calamagrostis epigejos* roots used for the statistical analyses.

| Site Age (y) | Spontaneous | | Reclaimed | | Total |
| --- | --- | --- | --- | --- | --- |
|  | Spring | Autumn | Spring | Autumn |  |
| 12 | 3 | 3 | 4 | 3 | 13 |
| 20 | 1 | 3 | 3 | 1 | 8 |
| 30 | 2 | 3 | 3 | 2 | 10 |
| 50 | 0 | 2 | 2 | 2 | 6 |
| Total | 6 | 11 | 12 | 8 | 37 |

**Table S3** Community matrix of operational taxonomic units of arbuscular mycorrhizal fungi.

Provided in separate file Table_S1_S3_S4_S5.xls, Sheet Table_S3

**Table S4** Community matrix of arbuscular mycorrhizal fungal taxa. Numbers are short sequence reads.

Provided in separate file Table_S1_S3_S4_S5.xls, Sheet Table_S4

**Table S5** Sub-sampled community matrix of arbuscular mycorrhizal fungal taxa.

Provided in separate file Table_S1_S3_S4_S5.xls, Sheet Table_S5

|  | Adj. R^2^ | F_statstics_ | *P* - value |
| --- | --- | --- | --- |
| *Fraxinus excelsior* | 0.063 | 3.43 | 0.004 |
| *Chamerion angustifolium* | 0.048 | 2.91 | 0.004 |
| *Pastinaca sativa* | 0.044 | 2.73 | 0.024 |
| *Acer pseudoplatanus* | 0.042 | 2.73 | 0.014 |
| *Deschampsia caespitosa* | 0.034 | 2.43 | 0.018 |

**Table S6** Plant species with significant relationship with AMF community composition (based on sub-sampled dataset).
