# Supplementary figures and images for: Plant Communities Rather than Soil Properties Structure Arbuscular Mycorrhizal Fungal Communities along Primary Succession on a Mine Spoil
Source: Front Microbiol. 2017 Apr 20;8:719. doi: 10.3389/fmicb.2017.00719 (PMC5397529; doi:10.3389/fmicb.2017.00719)

A

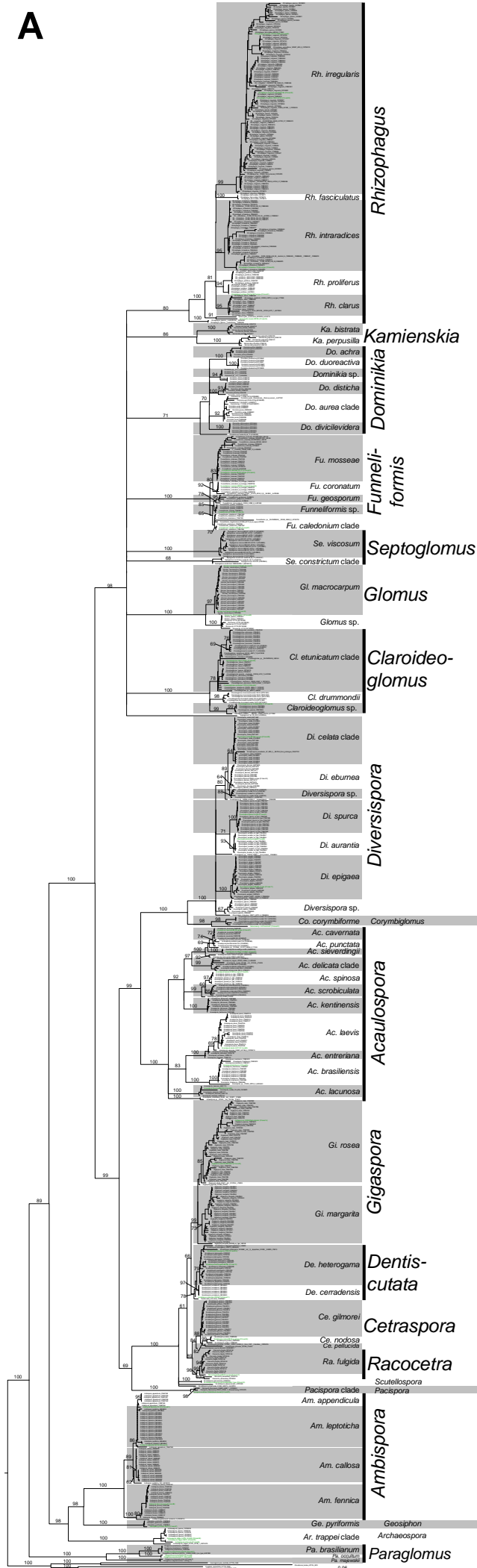

B

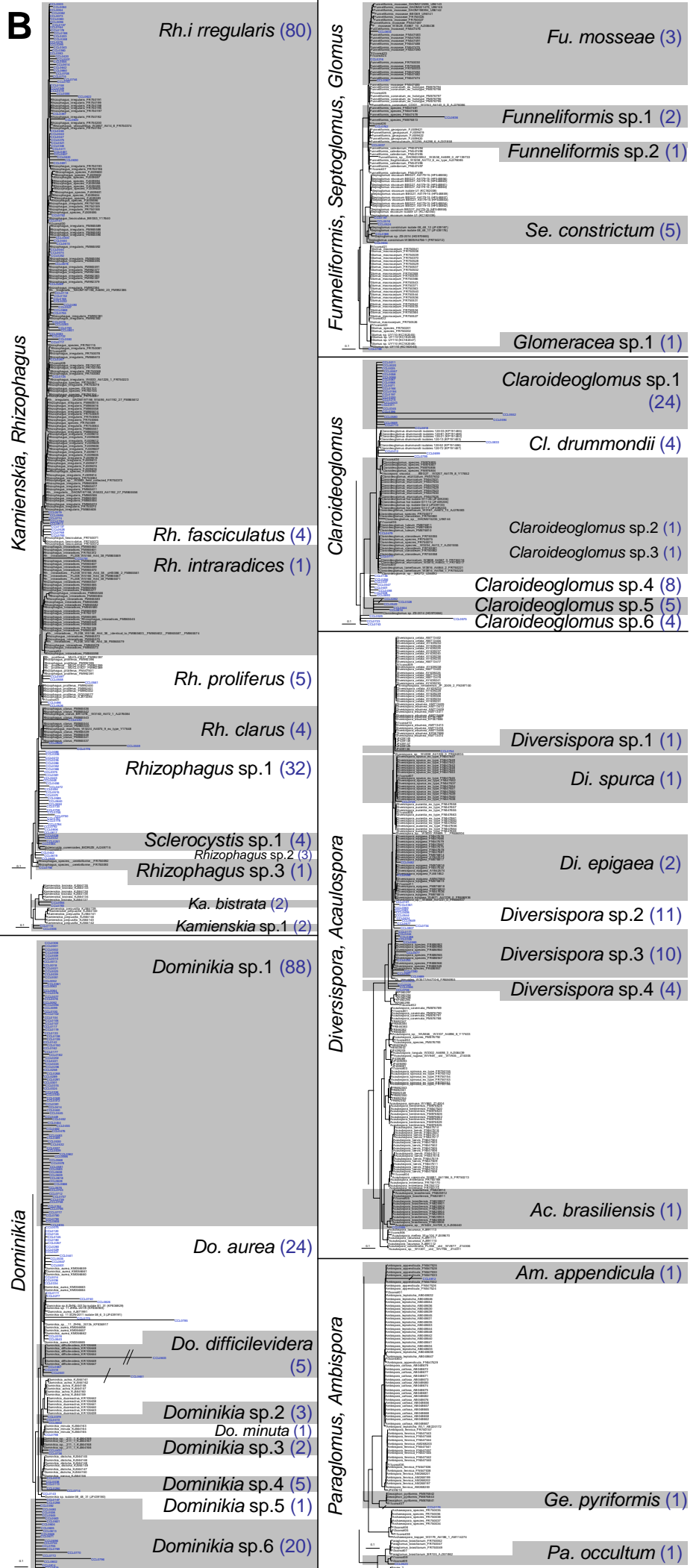

Supplement: Supplementary file 3 [file Image_2.pdf]
